# Real-Time Tunable Optofluidic Splitter via Two Laminar Flow Streams in a Microchannel

**DOI:** 10.3390/mi13101758

**Published:** 2022-10-17

**Authors:** Sha Xiong, Wenshuo Mai, Xiaofang Huang

**Affiliations:** School of Automation, Central South University, Changsha 410083, China

**Keywords:** optofluidics, splitter, microchannel, laminar flow, interface, refractive index

## Abstract

This paper reports a tunable optofluidic splitter in which the incident light is split via refraction and reflection at the interface between two laminar flows in a microchannel but with different refractive indices. A Y-junction microchannel is used to demonstrate the continuous tuning of the splitting ratio of optical power by smooth adjustment of the ratio of two flow rates. In addition, it has achieved the tuning of split angle from 5° to 19° by the control of the refractive index contrast. The dynamic response gives a fastest switching frequency of 1.67 Hz between the “wave-guiding” and “splitting” status.

## 1. Introduction

As a fundamental component, the optical beam splitter divides optical signals among different routes and has found broad applications in imaging [1,2], communication [3,4], photonic integrations [5] and biosensors [6,7]. In its most common form, the optical beam splitter is constructed by solid materials such as Wollaston prisms and half-silvered mirrors. However, traditional solid splitters have difficulties in meeting the demands of miniaturization and large tunability. Currently, microscale waveguide-based splitters are widely reported based on multimode interference (MMI) [7,8,9], photonic crystals [10] and plasmonic waveguides [11,12]. Although these technologies are capable of extreme miniaturization with low cost, they do not allow large tunability.

The last decades have seen rapid growth in developing optofluidic devices and their applications [13,14,15,16]. It brings a new class of adaptive optical components that can be integrated onto microfluidic chips. In optofluidic devices, optics can be built entirely out of liquids and the optical properties of the devices can be easily changed by manipulating the fluid flows. Different types of optofluidic devices have been demonstrated such as waveguides [17,18], lenses [19,20,21,22,23,24], gratings [25], prisms [26], switches [27,28] and attenuators [29,30]. The emerging of optofluidics has inspired the creation of a new type of “tunable” splitter. Wolfe et al. designed a microfluidic-based evanescent coupler including two liquid waveguides that share an inner cladding stream [17]. Ratios of the intensities of the light emitted from each waveguide are tuned by the flow rate of the inner cladding stream. Yang et al. introduced a transformation optofluidic Y-branch splitter based on the design of a spatially variable refractive index by controlling the convection–diffusion process of liquid-flowing streams [31]. The split angle is tuned by changing the flow rates and composition of the liquids. Li et al. demonstrated a 3D optofluidic Y-branch waveguide by introducing Dean flow [32]. The incident light is split at a Y-shape junction when two 3D liquid waveguides combine into one with a high flow rate. Although these optofluidic splitters can flexibly adjust the light intensity ratio or change the splitter angle, they need sophisticated control of the flow rates to achieve the desired refractive gradient index [31] or form multiple liquid waveguides (the number of flow streams ≥ 4) [17,32].

In this paper, we propose a different method to realize the optical splitting effect by use of the reflection and refraction of light at the optically smooth liquid interface between two laminar flows, which have different refractive indices, in a single microchannel. This optofluidic splitter has tunable splitting characteristics (e.g., the splitting ratio and split angle) conveniently controlled by manipulating two flow streams. 

## 2. Device Design and Working Principle

**Figure 1:**
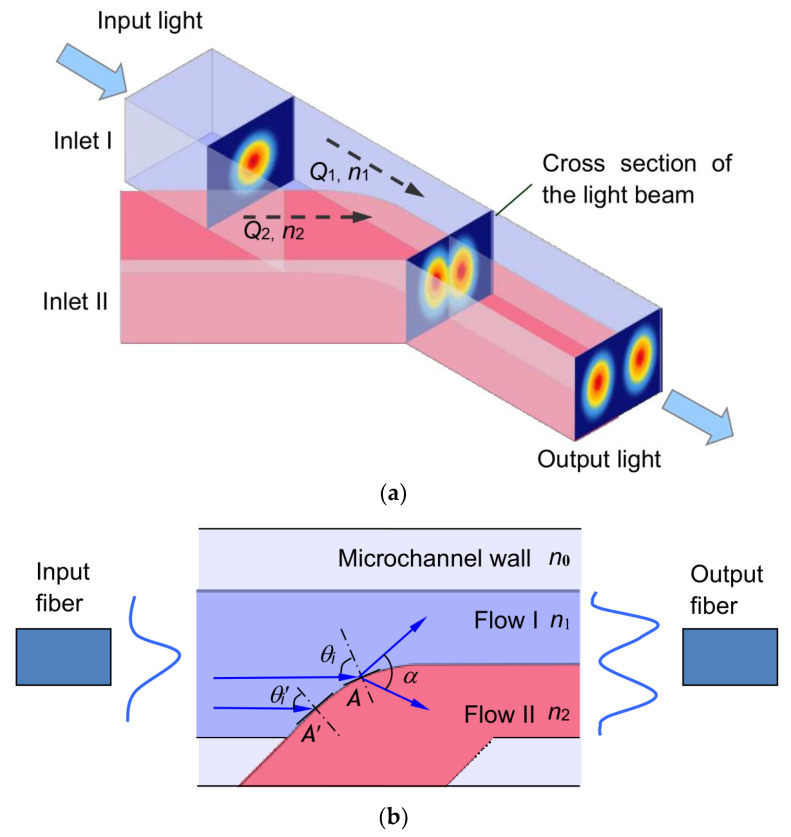
(**a**) Schematic of the optofluidic splitter in a microchannel. (**b**) Close-up of the intersection of two flows.

The schematic diagram of the optofluidic splitter is shown in Figure 1a. A Y-junction is formed by a main channel and a secondary channel with the same width *W*. Two flows (named flow I and flow II) with different refractive indices *n*_1_ and *n*_2_ merge at the intersection forming a curved interface. The incident light beam is injected from a single-mode fiber into flow I; it is then split into two light beams at the interface of the two flows (see Figure 1b). The optical splitting effect is due to the refraction and reflection of light with different incident angles at the curved interface. The splitting ratio of the optical powers in the two flows can be tuned by controlling the ratio of the two flow rates; the split angle (open-angle *α*) between the two light beams can be tuned by controlling the refractive indices of the two flows. As a result, a tunable optofluidic splitter is achieved.

The position of the interface can be solved from the Navier–Stokes equation [33] assuming that the viscosity ratio and flow rate ratio between the two flow streams are $\mu =\eta_{2}/\eta_{1}$ and $\varphi =Q_{2}/Q_{1}$, where the viscosity and flow rates are denoted by η and Q, respectively. The subscripts indicate flow I and flow II. The relative position of the interface can be estimated as

(1) $$\gamma =\frac{W_{1}}{W}=\frac{1}{1+\mu \varphi }$$

where *W*_1_ is the width of flow I. The optical splitting ratio should correlate with the width ratio of the two flows. Here, the optical splitting ratio is defined as $\delta =P_{1}/(P_{1}+P_{2})$, where *P*_1_ and *P*_2_ are the optical power of light in flow I and flow II, respectively. 

The refraction and reflection of the light at the interface can be approximated analytically by combining Snell’s law with the Fresnel equations [34]. The split angle *α* is the open angle between the reflected ray and the transmitted ray. For the ray with an incident angle *θ_i_*, the split angle *α* is given by

(2) $$\alpha =\underset{\alpha_{1}}{\underbrace{2\times (90{}^{\circ}-\theta_{i})}}+\underset{\alpha_{2}}{\underbrace{\left[\theta_{i}-\arcsin\left(n\sin\theta_{i}\right)\right]}}$$

where *n* = *n*_2_/*n*_1_, *n*_1_ and *n*_2_ are the refractive index of flow I and flow II, respectively. The light intensity changing with the incident angle should be considered. Split angle *α* can be divided into two parts: the deviation angle *α*_1_ between the incident ray and the reflected ray and the deviation angle *α*_2_ between the incident ray and the transmitted (or refracted) ray. For a given *n*, when the incident angle increases, the deviation angle *α*_1_ decreases and the deviation angle *α*_2_ increases. As the two flows make a curved interface, the normal of the interface is changed continuously. Thus, the incident angle would be different at different points of the interface even if all the incident light rays come along the horizontal direction. For example, the incident angle at point A is larger than that at point A’ as shown in Figure 1b. The intensity of the refracted light is decreased with the increasing incident angle. In this system, the transmittance is larger than 50% if the incident angle is smaller than 88°. According to the simulation results, the split angle is mainly determined by the grazing incidence between the two flows. Thus, the curvature of the interface at the junction has little influence on the split angle. Although the curvature of the interface is varied with the flow rates and the angle between two microchannels, the split angle is almost not affected by these flow conditions and geometrical parameters of the microchannels. In addition, the intensity of the refracted light must be larger than the detection limit of the camera. When the transmittance is less than *x*%, the transmission light cannot be detected by the camera; therefore the corresponding incident angle is ${\left.\theta_{i}\right|}_{T=x\%}$. However, the reflectance increases with the increasing incident angle, while *α*_1_ decreases with the increasing incident angle. That means *α*_1_ should be corresponding to the reflectance of 100%. Then

(3) $$\alpha =2\times (90{}^{\circ}-\theta_{i}\left|{}_{R=100\%})\right.+\left(\theta_{i}\left|{}_{T=x\%}\right.-\arcsin\left(n\sin\theta_{i}\left|{}_{T=x\%}\right.\right)\right)$$

It is well known that there is a linear relationship between the refractive index and concentration which can be expressed as [35]

(4) $$\Delta n=n_{z}-n_{x}=\frac{c}{\rho }\left(n_{y}-n_{x}\right)$$

where the *n_x_*, *n_y_* and *n_z_* are the refractive indices of the solvent, solute and solution, *ρ* is the density of the solute and *c* is the concentration in terms of weight of solute per unit volume of solution (*w*/*v*). Therefore, the split angle can be tuned by adjusting the concentration of the solution. On the other hand, the split angle can be used to evaluate the concentration of the solutions.

## 3. Experimental Results and Discussion

The microfluidic chip is fabricated with a standard soft lithography technique using polydimethylsiloxane (PDMS). The rectangular microchannel is 300 μm wide and 70 μm high. The Y-junction is formed by a main channel and a secondary channel with an injection angle of 45°. Deionized (DI) water and ethylene glycol aqueous solutions doped with Rhodamine 6G (0.01 mg/mL) were injected from the inlets by syringe pumps (Cavro XLP6000, Tecan, Männedorf, Switzerland). The refractive index of the ethylene glycol solution was changed from 1.333 to 1.430 by controlling the concentration of solutes. The refractive indices of the solutions were measured by the refractometer (AR200, Reichert, Buffalo, NY, USA). Light was coupled into the microchannel through a single mode fiber. An argon ion laser (488 nm) was used as the light source for exciting the Rhodamine 6G. The fluorescent images were captured using a charge coupled device (CCD) camera mounted on an inverted optical microscope (Eclipse Ti2-U, Nikon, Tokyo, Japan). The intensity of the light guided through the channel was measured from the output end of the microchannel. An output fiber connecting with the spectrometer (HR 4000, Ocean Optics Inc., Dunedin, FL, USA) was set at the end of the microchannel for the dynamic response measurement.

**Figure 2:**
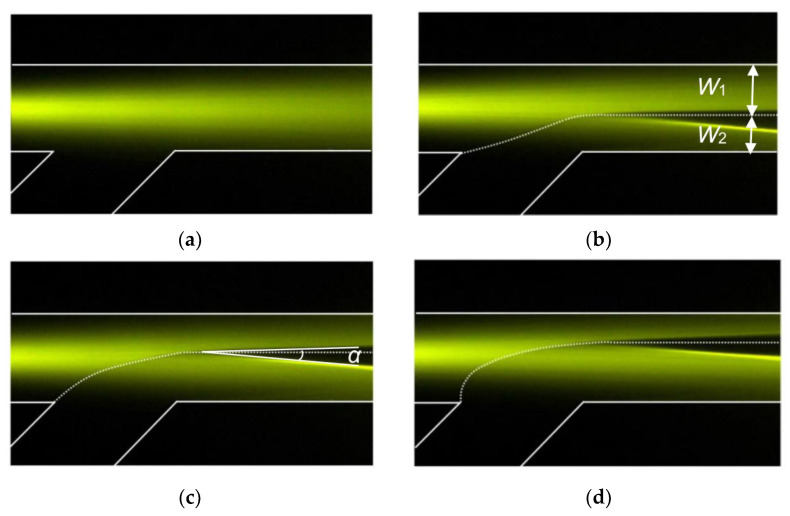
Beam splitter with different flow rate ratios: (**a**) 0, (**b**) 0.43, (**c**) 0.87 and (**d**) 1.5.

The top view of the microchannel is shown in Figure 2, in which the light propagation path is demonstrated clearly by the excited fluorescence. The refractive index of the microchannel wall (PDMS) is *n*_0_ = 1.412. The refractive indices of the ethylene glycol solutions in the two flows are *n*_1_ = 1.415 and *n*_2_ = 1.430. When only flow I was injected into the microchannel with a flow rate of 50 μL/min, the incident light was coupled into flow I and then propagated straight though the microchannel as shown in Figure 2a. When flow II was injected into the microchannel, the incident beam split into two beams. Figure 2b–d shows the observed splitting images when the flow rates are 35/5 (*Q*_1_ = 35 μL/min and *Q*_2_ = 15 μL/min), 27/23 and 20/30 μL/min, respectively. The corresponding flow rate ratio (*φ*) is 0.43, 0.87 and 1.5, respectively. It can be seen that the change of flow rates strongly affects the width of the flows and thus displaces the interface. As a result, the splitting ratio was modified. When *φ* was increased, the interface was pushed up and the width of flow II (*W*_2_) became larger; thus, more light was refracted into flow II. Another interesting observation is that the split angle is almost independent of *φ*. The split angle remains constant at approximately 7° during the adjustment of *φ*. Such a feature is desirable for practical applications as the splitting ratio and the split angle can be tuned independently.

**Figure 3:**
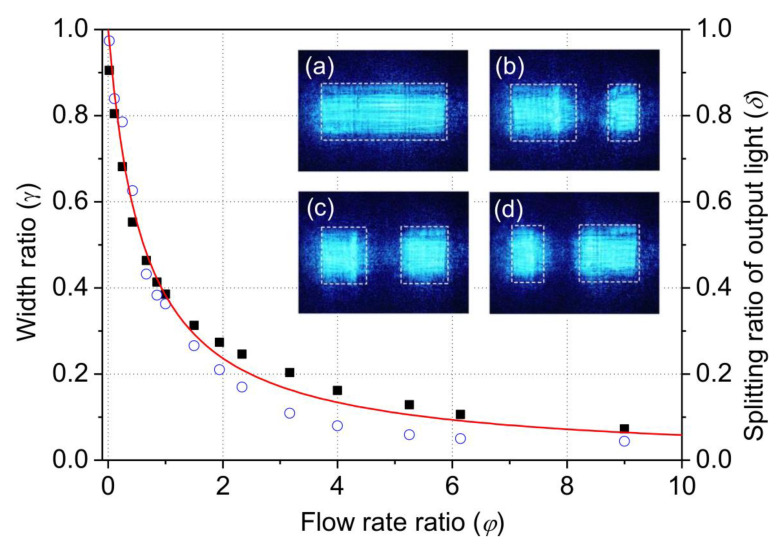
Relation between the width ratio (squares), splitting ratio of the optical power (circles) and the flow rate ratio. The solid line is the theoretical fitting. Inset: cross-sectional view of the light intensity distribution at the output end of the microchannel with different flow rate ratios (**a**) 0, (**b**) 0.43, (**c**) 0.87 and (**d**)1.5.

The relationship between the width ratio *γ* and flow rate ratio *φ* are shown in Figure 3. In the experiment, the total flow rate was fixed at 50 μL/min. *γ* decreased with the increasing *φ*. The experiment results agree well with the theoretical estimation based on Equation (1), which is depicted as the solid curve in Figure 3. For direct comparison with the width ratio, the splitting ratio of optical power (*δ*) is also plotted in Figure 3. The splitting ratio of optical powers is measured by summing up and comparing the intensities of the CCD pixels within the beam spot regions from the cross-sectional views of the output light as indicated in the inset of Figure 3. The splitting ratio of optical powers correlates well with the width ratio. The difference between the optical signal and the width ratio is caused by the Gaussian distribution of the input light. 

**Figure 4:**
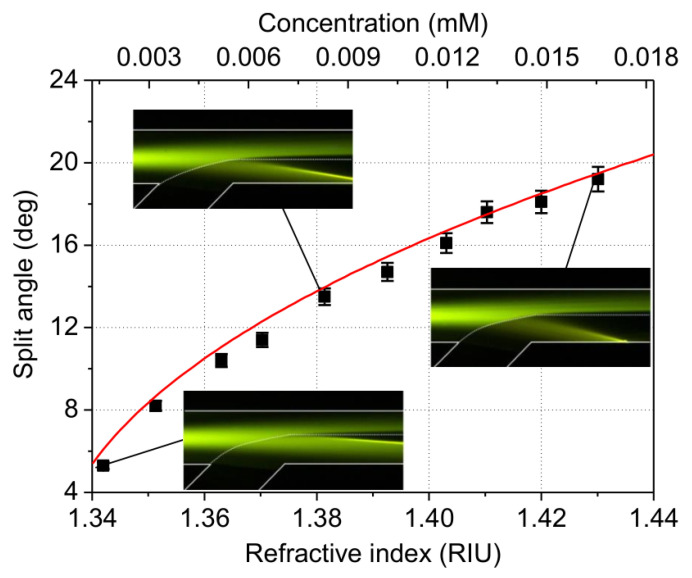
Split angles for given concentrations of ethylene glycol solutions in experiments. The squares are experimental data, solid line is theoretical prediction.

The measured split angle as a function of the refractive index is shown in Figure 4. Here, DI water was injected in flow I with *n*_1_ = 1.333 and different concentrations of ethylene glycol solution were injected in flow II with *n*_2_ changed from 1.342 to 1.430. Using a linear fit of the literature data [36], the refractive index of the solution was converted to concentration, which was changed from 0.00163 to 0.01654 mM. The split angle was increased from 5° to 19° correspondingly. By comparing the experimental results with the theoretical results from Equation (3), the threshold value of transmittance is 35%. This means that when the transmittance is less than 35%, the light rays cannot be detected. 

**Figure 5:**
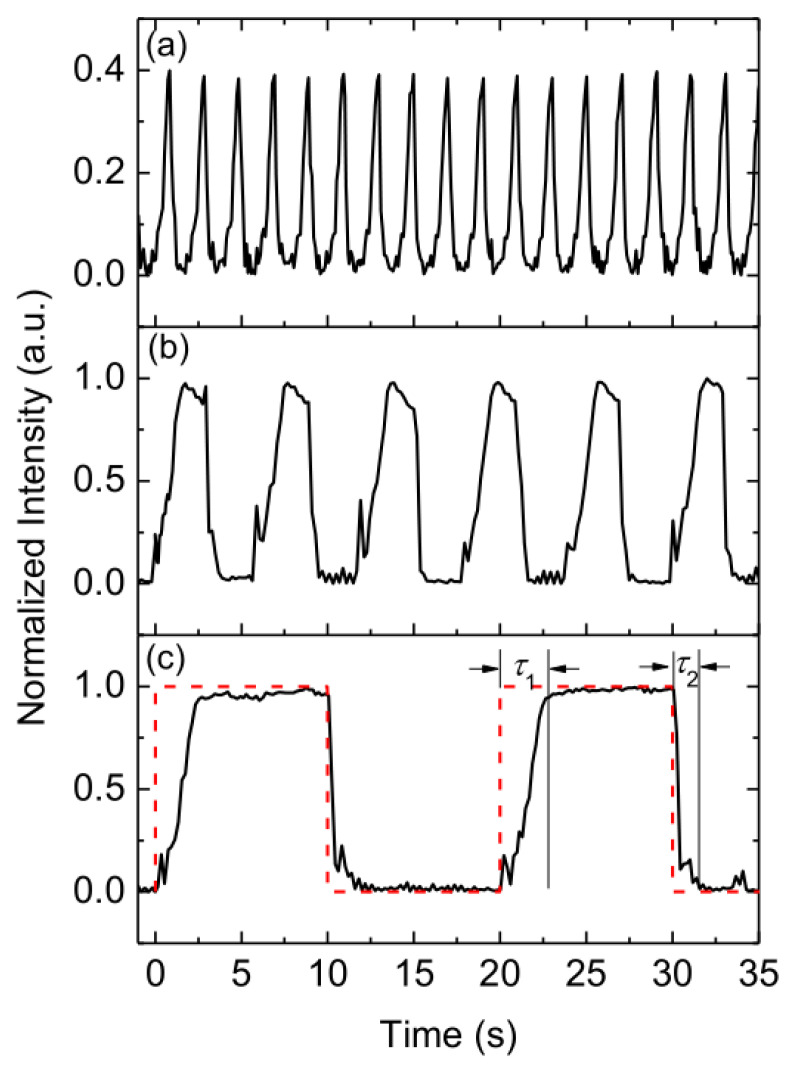
Detected signal with different switching periods: (**a**) 1 s, (**b**) 3 s, and (**c**) 10 s. *τ*_1_ is the rising time and *τ*_2_ is the falling time.

Figure 5 depicts the optical signals received from the output fiber, which demonstrates the dynamic response of the device (*n*_1_ = 1.415 and *n*_2_ = 1.430). When *Q*_1_ and *Q*_2_ were set to 50 and 0 μL/min respectively, the liquid waveguide was achieved and maximum intensity was detected. When *Q*_1_ and *Q*_2_ were both set to 50 μL/min, the optofluidic splitter was achieved and the minimum intensity was detected. There is a discrepancy between the rising time (2.74 ± 0.39 s) and the falling time (0.77 ± 0.20 s) as seen in Figure 5. This is caused by the buildup of backpressure in the pumping system [27]. Replacing the low-viscosity solution with a more viscous solution builds up a much lower backpressure than the reversed process. Therefore, the falling time is less affected by the viscosity difference. It is noticed that even the switching period is shorter than the response time; the switching signal can still be detected with a lower maximum intensity (as shown in Figure 5a). The limit of the switching period of the current experimental setup is approximately 0.3 s, which corresponds to the fastest switching frequency of 1.67 Hz.

The proposed method in this letter provides several advantages as compared to others. First, the fabrication involves only a single step using cheap polymer materials, which makes it suitable for rapid prototyping in lab-on-chip systems. Second, the optofluidic device has the benefit of easy and robust control due to its structural simplicity. Previous optofluidic devices, which had multi-layers [28] or multi-flows [17], used complex designs and complicated flow controls to change the optical path. The demonstrated two-flow device achieves real-time tunable splitting by controlling only the position of the interface between the flows. Third, the split angle is real-time tunable, which can be used to precisely adjust the distance between the output beams and made it compatible with other components on the chip. In addition, it can be switched between the “waveguide” and “split” modes, which achieves versatile components in the miniaturized systems.

## 4. Conclusions

In this paper, a tunable optofluidic splitter is designed, fabricated and characterized. The incident light beam is successfully split by the interface of two laminar flows in a microchannel. The splitting ratio (almost from 0 to 1) and split angle with a tunable range from 5° to 19° have been demonstrated to tune continuously and over wide ranges by controlling the flow rates and refractive indices. The dynamic response was evaluated by coupling out the optical signal in the middle of the microchannel. This optofluidic splitter utilizes a simple and cheap polymer soft-lithography technique and features a small size and large tunability, making it easy to integrate with other microfluidic components/systems for chemical and biochemical analytical applications. The splitter could provide two beams from a single light source for an on-chip interferometer with the tunable split angle enabling precise adjustment on the optical path. In addition, its sensitivity to the refractive index opens a new approach for the development of an on-chip refractometer.

## Data Availability

Not applicable.
